# CMV Infection in Pediatric Liver Transplantation and Comparison of Prophylaxis Methods Depending on Donor Serostatus

**DOI:** 10.5152/tjg.2025.24585

**Published:** 2025-11-07

**Authors:** Doğan Barut, Bora Kunay, Sema Yıldırım Arslan, Veysel Umman, Benan Dernek, Ezgi Kıran Taşçı, Gözde Akkuş Kayalı, Zümrüt Sahbudak Bal, Sema Aydoğdu, Funda Çetin, Miray Karakoyun

**Affiliations:** 1 Division of Gastroenterology, Hepatology, and Nutrition Disease, Department of Pediatrics, Ege University Medical School, İzmir, Türkiye; 2 Division of Infectious Disease, Department of Pediatrics, Ege University Medical School, İzmir, Türkiye; 3 Department of General Surgery, Ege University Medical School, İzmir, Türkiye; 4 Department of Pediatrics, Ege University Medical School, İzmir, Türkiye; 5 Department of Pediatrics, Sivas Numune Hospital, Sivas, Türkiye; 6 Department of Microbiology, Ege University Medical School, İzmir, Türkiye

**Keywords:** Antiviral treatment, CMV infection, liver transplantation

## Abstract

**Background/Aims:**

Cytomegalovirus (CMV) infection is a significant complication in pediatric liver transplant recipients. This study aimed to assess the incidence, risk factors, and suspected effects of CMV infection in children undergoing liver transplantation and compared acyclovir and ganciclovir-based preventive therapy.

**Materials and Methods:**

The study included 92 patients who had liver transplants at Ege University Transplantation Unit between 2011 and 2021. Sixty-four pediatric patients with preoperative CMV immunoglobulin G (IgG) positivity were analyzed. Patients with early mortality, re-transplantation within 6 months, and CMV IgG negativity were excluded.

**Results:**

The study consisted of 39 females (61%) and 25 males (39%), with a median age of 5.3 years at transplantation. Cytomegalovirus viremia occurred in 42 patients (65%), and CMV disease developed in 7 patients (10%). The median duration of CMV viremia was 40 days, and CMV disease was 105 days. Age was significantly associated with CMV disease development, with younger patients at higher risk (*P* = .007). The choice of antiviral treatment (acyclovir vs. valganciclovir) did not significantly impact the development of CMV viremia or disease.

**Conclusion:**

Cytomegalovirus viremia and disease are common in pediatric liver transplant recipients, with younger age being a significant risk factor for CMV disease. However, CMV viremia and disease did not significantly impact this cohort’s graft loss, acute cellular rejection, or mortality. The choice of antiviral treatment and immunosuppression protocols did not influence CMV outcomes. These findings highlight the need for vigilant monitoring and tailored management strategies for CMV in pediatric liver transplantation.

**Main Points**

- In Turkish children who have undergone liver transplantation and are immune to cytomegalovirus (CMV), the incidence of viremia is high, and the frequency of CMV disease is low.
- Younger patients, particularly those under 12 months, require careful monitoring due to their higher risk of CMV disease.
- Valganciclovir and acyclovir are viable options for CMV prophylaxis in intermediate-risk pediatric liver transplant recipients.

## Introduction

Cytomegalovirus (CMV), a herpesvirus, infects a significant portion of the global population, ranging from 60% to 100%. Cytomegalovirus is the primary source of opportunistic infection leading to clinical symptoms in children who have received an orthotopic liver transplant (OLT).^1^ The risk of CMV infection post transplant is closely linked to potent immunosuppressive treatments; rejection of the transplanted organ; and the presence of other viral, bacterial, or fungal diseases. This may happen as a result of the original infection spread by the donor allograft or the reactivation of a latent host virus.^2^ The CMV immunoglobulin G (IgG) serostatus of the donor and recipient significantly influences post-transplant CMV infection.

In addition, individuals may have bone marrow suppression and be susceptible to invasive conditions such as lymphadenopathy, hepatitis, pneumonia, colitis, gastritis, ulcers, pancreatitis, meningoencephalitis, and possibly fatal outcomes.^2^

Studies on reducing CMV infection in intermediate-risk OLT patients suggest frequent polymerase chain reaction (PCR) screening without antiviral use, though prophylactic antiviral therapy is more effective. Standard measures include intravenous ganciclovir, oral valganciclovir, and acyclovir at varying intervals post transplant.^3^^-^^6^

The 2013 guidelines issued by the American Association for the Study of Liver Diseases and the American Society of Transplantation state that patients who have undergone OLT and are at moderate risk of developing CMV infection (those who are CMV positive) should be given preventive treatment with valganciclovir or intravenous ganciclovir. Alternatively, it is recommended that patients undertake weekly CMV PCR surveillance for 3 months following transplantation. This approach aims to prevent CMV infection and its associated complications in this high-risk population.^2^ Previous studies did not compare antiviral regimens between D+/R+ and D−/R+ patients. Due to donor serostatus variations, CMV prophylaxis lacks specificity, requiring individualized risk-based strategies. This retrospective investigation examines the occurrence of CMV in liver transplant recipients who are either D+/R+ or D−/R+ and compares the effectiveness of valganciclovir and oral acyclovir in preventing CMV infection in the Turkish pediatric population.

## Materials and Methods

The investigation was conducted at a single center to examine pediatric liver recipients who underwent liver transplantation (LT) from deceased or living donors (OLTs) and were at a moderate risk for CMV infection. The period of the study was from January 2011 to December 2021. The study included patients who were CMV seropositive (R+) and lived for at least 30 days after transplantation. Patients who had received multiorgan transplants or had not received antiviral prophylaxis were excluded. All patients received a minimum of 12 months of follow-up. The medical data of 92 patients who underwent liver transplants between 2011 and 2021 were examined retrospectively. The occurrence of CMV infection in recipients of OLTs with donor-negative (D−/R+) or recipient-positive (D+/R+) CMV serostatuses and valganciclovir versus oral acyclovir effectiveness were compared in this study. The study targeted the pediatric transplant patients and sought to identify differences in CMV infection incidence between the 2 treatment groups. This project was approved by the Ege University Committee for Clinical Research Ethics on June 26, 2023, with decision no. 23-6.1T/2. The parents or legal guardians of patients provided signed informed consent.

### Cytomegalovirus DNA Detection and Quantification

Between 2011 and June 2018, PCR analyses were performed using the Abbott RealTime CMV test and the m2000 fully automated RealTime system (Abbott Molecular Inc., Des Plaines, ILLINOIS ). The analytical sensitivity of this assay ranges from 1.70 to 8.19 log IU/mL. From June 2018 to June 2019, CMV DNA isolation was conducted using the Anatolia Magnesia 2448 system (Anatolia Geneworks, Türkiye), and the extracted DNA samples were analyzed using the Bosphore® CMV Quantification Kit and the Montania 4896 real-time PCR thermal cycler (Anatolia Geneworks, Türkiye). The quantification range of this assay was 1.8 to 7.1 log IU/mL. Between June 2019 and September 2020, analyses were performed using the NeuMoDx CMV PCR Test (Quant Assay, Qiagen, Ann Arbor, USA) on the NeuMoDx™ 96 Molecular System platform. The dynamic range for this assay was 1.3 to 8.0 log IU/mL. From September 2020 to 2021, CMV DNA detection and quantification were performed using the cobas® CMV PCR test on the fully automated cobas® 6800 system (Roche Diagnostics, Mannheim, Germany). This assay had a dynamic quantification range of 1.54-7.00 log IU/mL.

### Serological Assays for Cytomegalovirus

Between 2011 and October 2018, CMV IgG and CMV IgM analyses were performed using the Architect CMV IgG and IgM Reagent Kit on the ARCHITECT i2000SR analyzer (Abbott Laboratories, Chicago, IL, USA). From October 2018 to 2021, analyses were conducted using the Alinity i CMV IgG and IgM Reagent Kit on the Alinity i® automated system (Abbott Laboratories, Chicago, IL, USA). For both the Architect and Alinity i CMV IgG assays, results were expressed in AU/mL, with non-reactive values defined as <6.0 AU/mL, equivocal/gray zone as 6.0 to <15.0 AU/mL, and reactive as ≥15.0 AU/mL. The calibrators covered the assay’s calibration range of 0.0 to 250.0 AU/mL. Throughout the study, CMV serology testing was performed on patient serum samples using chemiluminescent microparticle immunoassay technology, following the manufacturer’s instructions and employing the designated assay kits, timeframes, and analytical platforms.

### Immunosuppressive Protocol

The transplant team determined induction regimens at the time of transplant based on medical necessity. Induction regimens included corticosteroids and FK 506, which were used as standard immunosuppression agents after OLT. The primary approach to maintaining immunosuppression after transplantation relied on using calcineurin inhibitors (namely tacrolimus or cyclosporine), either alone or in conjunction with mycophenolate mofetil and steroids. Methylprednisolone (mPSL) was given intravenously; the dose was 10 mg/kg/day**, **on days 0 to 2 post-transplant, which was then reduced to 5 mg/kg/day on days 3 to 5. On day 6, the dosage was further decreased to 0.25 mg/kg/day. Following this initial phase, patients were transitioned to oral mPSL for continued treatment and tapered gradually according to the center’s protocol. Tacrolimus was started on day 1 after transplantation unless there was an exceptional issue, mainly regarding kidney function. Tacrolimus blood levels were aimed at 10-15 ng/mL for the 14 days after the transplant. The target level was approximately 10 ng/mL for 2 weeks. After the initial month, the target blood level was adjusted to 6-10 ng/mL. (7) The tacrolimus dose was individualized based on each patient’s blood concentrations and clinical course. This dose adjustment primarily considered the functional status of the transplanted liver to ensure optimal outcomes for each patient.

### Diagnosis of Cytomegalovirus Infection and Cytomegalovirus Disease

The American Society of Transplantation’s most recent recommendations for use in clinical trials^8^ provided the foundation for the definitions of CMV infection and illness.^8^ Cytomegalovirus infection was defined by detecting at least 1 pp65-positive cell or more than 250 copies/mL viral load in whole blood. CMV disease is characterized by CMV infection, clinical symptoms such as fever, malaise, and diarrhea, and signs like leukopenia and thrombocytopenia. It can also manifest as tissue-invasive CMV disease, which includes conditions like gastroenteritis, hepatitis, pneumonitis, or retinitis. Cytomegalovirus PCR monitoring was conducted on all liver transplant patients weekly for the first 3 months following the transplant, once every 2 weeks for the following 3 months, and once a month for up to a year. PP65 antigen/CMV PCR was performed to identify viremia during febrile episodes or leukopenia.

The co-occurrence of tissue-invasive CMV infection was the main research outcome measure. The frequency and severity of allograft rejection, patient and allograft survival, and an evaluation of the effectiveness of antiviral medication were among the other outcomes. The diagnosis and classification of acute rejection episodes were determined using the rejection activity index of the Banff schema.^9^

### Cytomegalovirus Prophylaxis

Patients were administered 7 × body surface area × creatinine clearance once daily (max dose of 900 mg) of valganciclovir; patients received oral acyclovir 600 mg/m^2^/dose (max dose of 800 mg, 3 times daily). Cytomegalovirus prophylaxis continued for 3 months post OLT. The patients who had CMV infection or disease received intravenous ganciclovir at a dosage of 5 mg/kg every 12 hours. Cytomegalovirus PCR was repeated 1 week after initiating ganciclovir therapy. Treatment continued for 2 additional weeks after the first negative CMV viral load was documented.

### Cytomegalovirus Monitoring Protocol

In this study, CMV viremia was monitored regularly according to a standardized protocol. Weekly CMV PCR testing was performed for the first 3 months post transplant, followed by bi-weekly testing for the next 3 months, and then monthly monitoring up to 1 year. Additionally, CMV testing was repeated immediately in cases of febrile episodes, leukopenia, or clinical suspicion of CMV infection. The clinical decisions based on these results included initiating or adjusting antiviral therapy when CMV DNAemia was detected and monitoring response to treatment with repeat PCR testing.

### Statistical Analysis

Categorical variables were compared using Fisher’s exact test, particularly in analyzing differences between patients with and without CMV viremia or CMV disease. Survival analysis was performed using the Kaplan–Meier method to estimate overall survival rates. To identify independent predictors of CMV viremia and CMV disease, a multivariate Cox proportional hazards model was applied, incorporating variables such as patient age, donor serostatus, antiviral prophylaxis type, and immunosuppressive regimen. Statistical analysis was performed using SPSS software version 18.0 (SPSS Inc.; Chicago, IL, USA).

## Results

**Figure 1.**
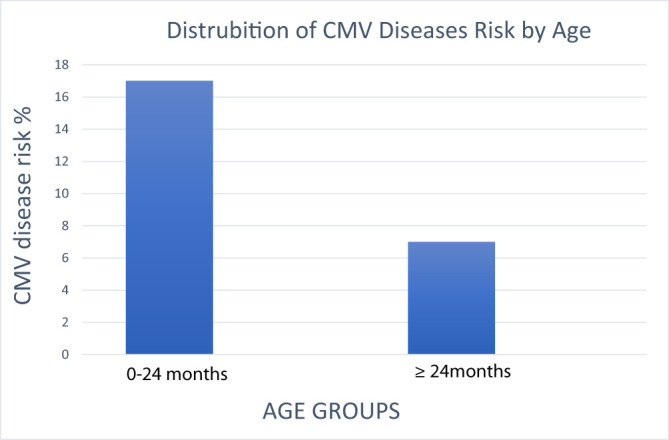
The distrubition of CMV disease by age group. CMV, cytomegalovirus.

A total of 92 patients underwent OLT between 2011 and 2021. Twelve patients had early period mortality, 8 had re-transplantation in the first 6 months, and 8 patients with CMV IgG negativity were excluded from the study (Figure 1). There were 39 female patients (61%) and 25 male patients (39%). At transplantation, the age ranged from 5 months to 19 years, with a median of 5.3 years. Forty-eight patients (75%) had liver transplantation with a living donor. Fifty-four (84%) patients were on tacrolimus, and 10 (16%) were on sirolimus treatment. Table 1 shows the etiology of liver transplantation in the patients. The CMV IgG positivity rates are also shown in Table 1.

**Figure 2.**
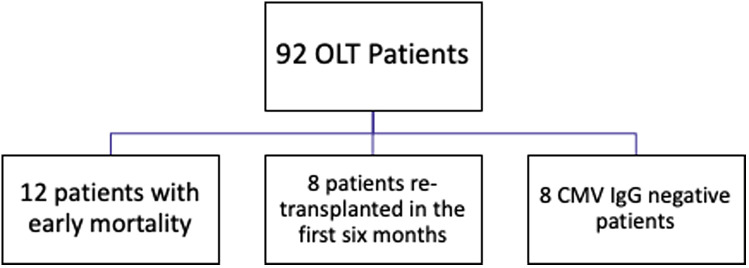
Study design. CMV, cytomegalovirus; OLT, orthotopic liver transplantation.

While there was no statistically significant age difference between patients with and without CMV viremia, younger age was significantly associated with the development of CMV disease (*P* = .007). Notably, infants under 12 months of age were disproportionately affected, highlighting the need to interpret CMV risk in pediatric liver transplant recipients according to age rather than treating them as a homogeneous group. This age-dependent vulnerability suggests a requirement for more intensive surveillance and earlier intervention thresholds in younger patients. It was discovered that CMV disease increased in frequency as age decreased, which was statistically significant. The distribution of CMV disease by age group is shown in Figure 2. When both the CMV viremia group and the CMV disease group were compared according to gender, a statistically significant difference did not exist (Table 2).

We randomly preferred acyclovir treatment in 36 patients (56.3%) and valganciclovir treatment in 28 patients (43.7%). During the initial years of the study period, from 2011 to 2016, acyclovir was more frequently utilized, being administered in 30 out of 36 cases. In contrast, after 2017, there was a notable shift toward the use of valganciclovir, which was prescribed to 22 out of 28 patients.

In 24 patients under acyclovir treatment and 18 patients undergoing valganciclovir treatment, there was CMV viremia; however, there was no correlation between the choice of acyclovir or valganciclovir on the development of CMV viremia (*P* = .392). The demographic characteristics of patients receiving valganciclovir and acyclovir were also analyzed. Table 3 shows the correlation between CMV viremia/disease in individuals who were treated with acyclovir and valganciclovir.

The development of CMV disease was not influenced by the decision for 2- or 3-drug immunosuppressive regimes. Table 4 shows the relationship between the choice of immunosuppression protocol, duration of steroid treatment, and the presence of CMV disease. Table 5 shows an overview of the demographic and clinical features of individuals diagnosed with CMV disease.

## Discussion

This retrospective study examined pediatric liver transplant recipients over a 10-year period, focusing on the incidence and management of CMV infection and disease. Younger age was significantly associated with an increased risk of CMV disease (*P* = .007), but no significant gender difference was noted. Patients receiving valganciclovir (n = 28) and acyclovir (n = 36) exhibited similar rates of CMV viremia (64% vs. 66%, *P* = .392) and CMV disease (7% vs. 13%, *P* = .349). No significant correlation was found between CMV infection and graft loss (*P* = .635), ACR (*P* = .385), or mortality (*P* = .335).

These findings contribute to the understanding of CMV infection dynamics in this vulnerable population and offer insights into the efficacy of different prophylactic treatments and immunosuppressive protocols. The study investigation in pediatric patients suggests that short-term prophylaxis and preemptive therapy could be a viable approach for long-term prophylaxis to prevent CMV infection. The incidence of symptomatic CMV disease has been low (10%), with no deaths or graft loss due to CMV.

There is insufficient research on the frequency of CMV disease and viremia, as well as the appropriate length of antiviral prophylaxis in pediatric liver transplant patients. An analysis of 155 children who underwent liver transplantation and were given induction immunosuppression using interleukin-2 receptor or anti-lymphocyte treatment revealed a 19.8% overall incidence of CMV disease. However, the study did not provide details on the methods used for CMV surveillance, prevention, or treatment.^10^ A limited retrospective study investigated the administration of valganciclovir for a duration of 100 days after surgery in 10 children who underwent liver transplantation. The study observed a single instance of CMV viremia occurring during preventive treatment. The research did not state the duration of follow-up or the ultimate incidence of CMV disease, and none of the recipients were CMV-positive/donor-negative matches.^11^ The CMV infection rates between patients receiving valganciclovir and those receiving acyclovir were analyzed. This study found no statistically significant difference in CMV viremia or CMV disease incidence between the 2 groups (*P* = .392 and *P* = .349, respectively). Demographic characteristics, including age and underlying disease, were similar between the 2 treatment groups. However, valganciclovir use increased in later years of the study period, with a shift in preference from acyclovir to valganciclovir after 2017.

The study did not reveal any significant age difference between the viremia group and those without. However, it did find that CMV disease was more frequent in younger patients. This suggests that risk stratification based on the mismatch of CMV serostatus may not be an effective predictor of CMV infection after liver transplantation in children. This could be due to the passive transfer of maternal CMV antibodies in children who lack effective CMV-specific antibodies until 12-18 months old. The presence of passive maternal CMV antibodies in young children complicates the assessment of CMV serostatus and, consequently, the risk evaluation of CMV infection after pediatric liver transplantation. Cytomegalovirus-immune pediatric liver transplant recipients should have different follow-up protocols based on their age. Since infants younger than 12 months of age are at higher risk of transition from viremia to clinical CMV disease, this group should be subjected to more frequent viral load monitoring, and preemptive treatment initiation with lower thresholds should be considered. Infants are known to be at higher risk of CMV and other viral infections following liver transplantation, so many guidelines emphasize the importance of developing strategies to assess risk levels in this vulnerable population. This challenge necessitates a nuanced approach to managing and preventing CMV infection in pediatric liver transplant recipients, especially in newborns.^12^^,^^13^

In this study, OLT patients with an intermediate risk of CMV infection were looked at. It was noticed that using oral valganciclovir as a preventative strategy was just as effective as using acyclovir in people with CMV viremia to prevent CMV disease. In addition, it was found that the use of acyclovir/valganciclovir in patients with CMV disease did not affect the development of CMV disease. In this study, the incidence of CMV disease was notably lower than previously documented rates among OLT recipients at intermediate risk for CMV infection. This suggests that the D−/R+ and D+/R+ groups have a lower risk of CMV infection due to their pretransplant immunity to the virus, protecting against CMV infection transmitted through donor organs. After a retrospective analysis of 117 R+ OLT recipients, Singh et al^3^ reported that 38 (32.5%) had CMV disease develope without antiviral therapy. Additional examination of R+ patients revealed that the occurrence of CMV infection was notably higher in those who had a transplant from donors who tested positive for CMV (73.7%) compared to those who received a transplant from donors who tested negative for CMV (45.6%; *P* = .005). This finding highlights the significant impact of the donor’s CMV serostatus on the risk of infection among R+ OLT recipients.^3^ Kim et al found that 55.7% of patients who were positive for D+ and R+ acquired CMV infection during 13 years (n = 618). However, the researchers did not consider whether the patients received antiviral prophylaxis, which might have affected the actual occurrence of the illness. A controlled trial compared the effectiveness of oral ganciclovir prophylaxis and oral acyclovir in preventing CMV disease in intermediate-risk OLT recipients. The study indicated that oral ganciclovir prophylaxis was more effective than oral acyclovir. During the first year following OLT, the incidence of CMV disease in R+ patients treated with ganciclovir was 0.9%, whereas the incidence was notably higher at 7.3% among patients who received oral acyclovir. The choice of prophylactic agent should consider other factors such as side effect profiles, patient tolerance, and cost.

The potential indirect effects of CMV viremia or disease on LT outcomes were assessed. In this cohort, children who developed CMV viremia or disease did not experience an increase in acute cellular rejection episodes or graft loss, with an overall incidence of about 14% to 9%. In this study, none of the other complications, such as biliary, vascular problems, or graft loss, were found to be related to CMV status or infection. These outcomes are consistent with several adult and pediatric studies, suggesting that with effective management, CMV infection does not necessarily translate to poorer graft or patient survival.^15^ A significant trend in CMV infection rates or mortality was not observed across different years.

In this study, the preference for 2-drug immunosuppression protocols or the 3-drug immunosuppression protocol did not affect the onset of CMV disease, and the level of immunosuppressive treatment was also compared in CMV disease-absent and present cases, with no statistically significant difference between the 2 groups. The correlation between the duration of steroid use and the development of CMV disease was also analyzed, and no statistically significant difference could be found. This finding supports the current practice of tailoring immunosuppression to individual patient needs without heightened concern for increased CMV disease risk. In liver transplant patients, calcineurin inhibitors (CNIs) such as tacrolimus and cyclosporine constitute the cornerstone of immunosuppressive therapy. To mitigate CNI-associated side effects, current strategies focus on minimizing CNI use while combining it with anti-metabolites (such as mycophenolate and mycophenolate mofetil [MMF]) and steroids. This approach aims to achieve adequate immunosuppression while reducing the potential for toxicity and complications associated with prolonged CNI use. Cytomegalovirus infections are aggravated by MMF, which results in contrary and less beneficial consequences.^16^ mTOR: Mammalian target of rapamycin mTOR inhibitors have appeared as a promising option to MMF in strategies that aim to minimize the use of CNIs.^17^ Numerous large-scale meta-analyses have demonstrated that the use of mTOR inhibitors in various types of solid organ transplantation leads to a 2- to >3-fold reduction in CMV replication compared to MMF. Additionally, transitioning from MMF to mTOR inhibitors as an adjunctive therapy to tacrolimus improves CMV infection-free survival.

The findings of this study contribute valuable insights into CMV management in pediatric liver transplant recipients. One of the strengths of this study is the comprehensive comparison of 2 commonly used prophylactic agents—valganciclovir and acyclovir—demonstrating their comparable efficacy in preventing CMV viremia and disease. Furthermore, the structured analysis of patient demographics and treatment response provides clarity on factors influencing CMV outcomes.

However, several limitations should be considered:

The retrospective nature of the study limits the ability to control for confounding variables, such as variations in immunosuppression regimens or differences in adherence to prophylaxis protocols.

The study population is limited to a single center, which may affect the generalizability of the results.

Long-term outcomes beyond the first years post-transplantation were not analyzed in detail.

Despite these limitations, the results suggest that both valganciclovir and acyclovir remain viable options for CMV prophylaxis in intermediate-risk pediatric liver transplant recipients. Future prospective studies with larger cohorts and longer follow-up periods are needed to validate these findings and optimize prophylaxis strategies.

This study underscores the importance of individualized CMV prophylaxis and management strategies in pediatric liver transplant recipients. While CMV viremia is relatively common, progression to symptomatic CMV disease can be effectively controlled with current prophylactic measures, regardless of whether acyclovir or valganciclovir is used. Younger patients, particularly those under 12 months, require careful monitoring due to their higher risk of CMV disease. Immunosuppressive protocols should continue to be individualized, focusing on minimizing adverse effects while maintaining efficacy. Further research is needed to optimize CMV management in this population and to refine strategies for those at highest risk.

## Figures and Tables

**Table 1.** Etiologies for Transplantation and Cytomegalovirus Immunoglobulin G Positivity Rate

	n (%)
**Etiology**	
Biliary atresia	15 (23)
Metabolic disease	14 (22)
Fulminant hepatitis	11 (18)
Hepatoblastoma	10 (15)
Autoimmune hepatitis	3 (5)
Intoxication (paracetamol, iron)	4 (7)
Others	7 (10)
**CMV IgG Status**	
D+ / R+	52 (81)
D− / R+	2 (3)
D? /R +*	10 (16)

D+ CMV IgG / R+ CMV IgG, D− CMV IgG / R+CMV IgG.

CMV, cytomegalovirus; D, donor; IgG, immunoglobulin G; n, number of patients; R, recipient.

*The donor CMV IgG levels of 10 patients undergoing cadaveric liver transplantation could not be evaluated.

**Table 2.** Comparison of Patient Characteristics by Cytomegalovirus Viremia and Disease Status

	CMV viremia (+) N = 42	CMV disease (+) N = 7	*P*
Sex (female), n (%)	30 (71.4)	5 (71)	.311
Age at transplatation (median, Q1-Q3)	5.925 2-10	3.87 ± 3.85 1-6	.247
CMV IgG status, n (%) D+ / R+ D− / R+	40 (95) 2 (5)	7 (100) 0 (0)	NA
Age at CMV viremia/disease (median, Q1-Q3)	2, 1-8	2, 2-4	.007
Underlying disease, n (%) Biliary atresia Metabolic disease Fulminant hepatitis Malignancy Autoimmune disease Toxic hepatitis Other (Alagille syndrome)	9 (21.4) 8 (19) 8 (19) 8 (19) 2 (5) 2 (5) 5 (12)	3 (57.1) 1(14) – 1 (14) 1 (14) – 1	
Rejection, n (%) Acute cellular rejection Graft loss, n (%)	7 (16) 5 (12)	1 (14) 1 (14)	.385 .635
Immunosuppressive treatment, n (%) Tacrolimus Sirolimus	36 (85.8) 6 (14.6)	5 (71.5) 2 (28.5)	.436 .268
Peak tacrolimus levels at first month (mean ± SD) (mg/dL)	13.84 ± 4.5	14.88 ± 2.47	.114
Tacrolimus levels before CMV viremia /disease (mean ± SD) (mg/dL)	8 ± 2.84	11.8 ± 2.38	.600
Acyclovir, n (%)	24 (57)	5 (71)	.392
Valganciclovir, n (%)	18 (43)	2 (28)	.349
Mortality, n (%)	5 (12)	1 (14)	.335

*P*-values less than .05 indicate statistical significance.

CMV, cytomegalovirus; D, donor; IgG, Immunoglobulin G; n, number of patients; *P*, *P*-value; R, recipient.

**Table 3.** The Relationship Between Acyclovir/Valganciclovir Treatment and the Presence of Cytomegalovirus Viremia/Disease

	Acyclovir treatment n = 36 (%)	Valganciclovir treatment n = 28 (%)	*P*
CMV viremia, n (%)	24 (66)	18 (64)	.392
CMV disease, n (%) Hepatitis Gastroenteritis	5 (13) 4 1	2 (7) 2 0	.349
Male	22 (61)	17 (60)	.311

*P*-values less than .05 indicate statistical significance.

CMV, cytomegalovirus; n, number of patients; *P*, *P*-value.

**Table 4.** The Relationship Between Choice of Immunosuppression Protocol, Level of Immunosuppression, and Duration of Steroid Treatment with the Presence of Cytomegalovirus Disease

	CMV Disease Absent N = 57	CMV Disease Present N = 7	*P*
Two-drug immunosuppression (steroid and calcineurin inhibitor-based), n (%)	49 (86)	6 (85)	.673
Three-drug immunosuppression (steroid, calcineurin inhibitor, mycophenolate mofetil), n (%)	8 (14)	1 (15)	
Level of immunosuppression (tacrolimus), mg/dL (median, Q1-Q3)	12, 4-16	12, 6-15	.317
Duration of steroid treatment, month (median, Q1-Q3)	8,4-10	8, 2-12	.243
Mortality	5	1	.335

CMV, Cytomegalovirus; n, number of patients; *P*, *P*-value.

**Table 5.** Clinical Information on 7 Cytomegalovirus Disease Patients

Patients	Age (Months)	Sex	Donor type	Diagnosis	CMV Serostatus	Time to Onset After Transplant (Months)	Chronic Viral Load	CMV Symptoms and Signs	Immunosuppression Regimen	Duration of Steroid Treatment (Months)	Antiviral Regimen	Allograft Rejection / Graft Failure	Mortality
1	72	F	Cadaveric	Wilson	D+ / R+	3	303.892	Fever, hepatitis	Three-drug immunosuppression (steroid, calcineurin inhibitor, MMF)	8	Acyclovir	−/−	–
2	6	F	Living, father	Biliary atresia	D+ / R+	2	8.175	Fever, hepatitis	Two-drug immunosuppression (steroid and calcineurin inhibitor-based)	9	Acyclovir	−/−	–
3	6	M	Living, father	Biliary atresia	D+ / R+	12	46.687	Fever, hepatitis	Two-drug immunosuppression (steroid and calcineurin inhibitor-based)	12	Acyclovir	−/−	–
4	24	M	Living, uncle	Alagille	D+ / R+	3	1.570	Fever, hepatitis	Two-drug immunosuppression (steroid and calcineurin inhibitor-based)	10	Acyclovir	+/−	+
5	27	F	Living, father	Hepatoblastoma	D+R+	4	576.000	Fever, hepatitis	Two-drug immunosuppression (steroid and calcineurin inhibitor-based)	7	Acyclovir	−/+	–
6	6	F	Living, father	Biliary atresia	D+R+	3	3.813	Fever, hepatitis	Two-drug immunosuppression (steroid and calcineurin inhibitor-based)	8	Valganciclovir	−/−	–
7	32	M	Cadaveric	Autoimmune hepatitis	D+, R+	6	9.060	Fever, gastroenteritis	Two-drug immunosuppression (steroid and calcineurin inhibitor-based)	8	Valganciclovir	−/−	–

CMV, cytomegalovirus; D, donor; F, female; M, male; MMF, mycophenolate mofetil; R, recipient.

## Data Availability

The data that support the findings of this study are available on request from the corresponding author.
